# Serum bile acids and risk factors for colorectal cancer

**DOI:** 10.1038/sj.bjc.6601608

**Published:** 2004-02-03

**Authors:** A van Faassen, A Tangerman, B H Bueno-de-Mesquita

**Affiliations:** 1 Department of Urology, University Hospital, PO Box 5800, Maastricht 6202 AZ, The Netherlands; 2 Department of Medicine, Division of Gastroenterology, University Medical Centre St. Radboud, PO Box 9101, Nijmegen 6500 HB, The Netherlands; 3 Department of Chronic Disease and Environmental Epidemiology, National Institute of Public Health and the Environment, PO Box 1, Bilthoven 3720 BA, The Netherlands

**Keywords:** bile acids, diet, colorectal cancer

## Abstract

The ratio of deoxycholic acid to chenodeoxycholic acid in the serum of 62 men was inversely related to body mass index and to saturated fat intake after adjustment for body mass index, smoking, and age conversely, this ratio was associated positively with the intake of fibre from grains.

Bile acids (BA) are potential risk factors for colorectal cancer (CRC). A small prospective study suggests that a high ratio of deoxycholic acid (DC) to cholic acid (CA) in serum is associated with an increased incidence of CRC (Costarelli *et al*, 2002). Based on a complete model of BA metabolism (Chaplin, 1998), it can be argued that the ratio of unconjugated DC to unconjugated chenodeoxycholic acid (CDC) in serum, in short the DC/CDC ratio, is relevant to CRC. Unconjugated DC in serum is regarded as being a biomarker for habitual colonic exposure to the cancer-promoting DC (Bayerdörffer *et al*, 1993), because the unconjugated BA is absorbed in the colon. The DC/CDC ratio may be a marker with high intraindividual reproducibility, because the colonic absorption of unconjugated DC is nearly equal to that of unconjugated CDC (Mekhjian *et al*, 1979) and the hepatic clearance of DC from the portal vein is equal to that of CDC (Ahlberg *et al*, 1977). The only difference between the variation of DC and that of CDC can be attributed to the dehydroxylation of the primary bile acid cholic acid (CA) to the secondary bile acid DC. The formation of CA, unlike that of CDC, is regulated by the amount of BA returning to the liver. If less unconjugated DC is returned, relatively more CA will be formed and the DC/CDC ratio in serum will be higher.

We studied the relation between the DC/CDC ratio in serum and such CRC-promoting (dietary) factors as intake of energy, saturated fat, fibre, calcium, smoking and body mass index (Giovannucci, 2003).

## MATERIALS AND METHODS

Nonfasting blood specimens were collected from 121 men and women in the range of 20–60 years old, who participated in the validation study of the Dutch food frequency questionnaire for the European Prospective Investigation into Cancer and Nutrition (EPIC) (Ocké *et al*, 1997). The samples of the 62 men, from which the blood was drawn more than 4 h after a meal, were selected for analysis of individual BA in the serum, as described before (Salemans *et al*, 1993). Only men were selected for logistic reasons.

## RESULTS

The characteristics of the study subjects, the dietary intakes of nutrients and the concentrations of total unconjugated BA in serum are shown in Table 1

**Table 1:** Characteristics of 62 men, their daily dietary intake and serum bile acid concentrations

	**Mean**	**s.d.**	**Min**	**Max**
Age (years)	43.7	11.2	22.9	60.5
BMI (kg m^−2^)	25.66	2.77	20.85	33.14
Total unconjugated bile acids in serum (*μ*mol l^−1^)	0.80	0.91	0.09	5.99
Ratio deoxycholic acid to chenodeoxycholic acid in serum	1.16	0.98	0.03	5.14
Daily dietary intake energy (MJ)	10.6	2.6	2.7	17.0
Saturated fat (g)	39.1	12.5	16.7	68.4
Calcium (mg)	1160	445	450	2900
Dietary fibre (g)	25.4	2.8	13.3	41.1
Dietary fibre from vegetables (g)	5.3	2.4	1.0	12.4
Dietary fibre from fruits (g)	3.3	2.1	0.2	8.2
Dietary fibre from other sources (g)	16.8	5.0	4.9	29.8

. Dietary fibre from sources other than fruits and vegetables was derived mainly from grains. In all, 25% of the men were current smokers.

Saturated fat intake was inversely related to the log of serum DC/CDC ratio univariately, as well as after adjustment for potential confounding factors like body mass index, smoking age and (Table 2

**Table 2:** Multivariate regression of log DC/CDC with dietary variables

	**β**	***R*^2^**
Saturated fat (g d^−1^)	−0.019**	0.19
Adjusted 1 # except energy intake		
Calcium (mg d^−1^)	−0.0006**	0.09
Total dietary fibre (g d^−1^)	0.048**	0.27
Adjusted 1		
Dietary fibre from other sources (g d^−1^)	0.050*	0.25
Adjusted 1		

# adjusted 1: adjusted for potential confounders (age, smoking, body mass index and energy intake).

* *P*<0.1;

** *P*<0.05.

). Also, calcium intake was inversely associated to the log of serum DC/CDC ratio in univariate analyses, but disappeared in the adjusted models. Conversely, for total dietary fibre, a positive relation was observed in the multivariate model, due to dietary fibre from sources other than fruits and vegetables. Significant associations were observed neither with the absolute serum levels of DC nor with those of CDC. In the full model, the dietary factors and potential confounders explained 29% of the variance in DC/CDC ratio. Smokers had a lower DC/CDC ratio than nonsmokers, but the difference was not statistically significant. The correlation between the DC/CDC ratio and body mass index was −0.28 (*P*<0.05).

## DISCUSSION

Serum BA is an attractive biomarker of CRC. However, the relations between serum BA and (dietary) factors related to CRC-risk *have not been studied*. We found an inverse relation between the DC/CDC ratio and the intake of saturated fat, and a positive relation with the intake of dietary fibre from grains. The relation with body mass index was negative. All these relations can be explained by the complete model of BA metabolism.

The significant negative correlation between DC/CDC in the serum and intake of saturated fat can be explained by the model of BA metabolism, taking into account the effect of saturated fat on faecal pH. Faecal pH is lowered by a high intake of saturated fat in humans (Gregoire *et al*, 1991), and a high intake of saturated fat leads to a higher concentration of saturated fatty acids in the intestine (Brussaard *et al*, 1983), which will bind almost all calcium in the intestine. This will leave intestinal phosphate to be absorbed. Less phosphate in the colon means less buffering of the acid-producing fermentation of fibre and a lower colonic pH. Long-chain saturated fatty acids bind calcium more strongly than long-chain unsaturated fatty acids (Cheng *et al*, 1949). A lower colonic pH results in less formation of DC from CA, while the activity of the bacterial enzyme 7*α*-dehydroxylase is inhibited *in vitro* at pH<6 (MacDonald *et al*, 1978).

The positive relation between DC/CDC ratio and dietary fibre from sources other than fruits and vegetables can be explained by the latter being mainly derived from grains that contain wheat bran and resistant starch. These will lower the concentrations of BA in the intestine due to increased faecal wet weight (Cummings *et al*, 1978; Heijnen *et al*, 1998), and therefore lower the concentrations of BA returning to the liver. The synthesis of CA and therefore the formation of DC will be less depressed.

Obese subjects have higher faecal bile acid concentrations (Miettinen, 1976), which will lead to less synthesis of CA and therefore to a lower DC/CDC ratio in serum. Other factors influence the value of DC/CDC ratio. In our previous study (Van Faassen *et al*, 1997), 20% of the variance in DC/CDC ratio in men (*n*=16) could be explained by the pH of foecal water (*β*=0.28±0.40), defecation frequency (*β*=0.80±0.48) and log DC in faecal water (*β*=0.30±0.70).

The consistency in the direction of the relation between the DC/CDC ratio and risk factors for CRC compared to that of the direction of the relation between the risk factor and CRC risk (Table 3

**Table 3:** Relation between DC/CDC and risk factors for CRC (this study) and the relation between the risk factors and CRC risk (literature)

**Risk factor**	**DC/CDC**	**CRC-risk**	**Reference**
Saturated fat	−	+	Giovannucci (2003)
Fibre from grains	+	−	Giovannucci (2003)
Body mass index	−	+	Giovannucci (2003)

) leads to the hypothesis that a high CRC risk may be associated with a low DC/CDC ratio in serum. Until now, this has been found neither in prospective (Costarelli *et al*, 2002) nor case–control studies (Bayerdörffer *et al*, 1993). Unconjugated individual BA will have to be analysed and several confounding factors have to be examined, such as the time of blood collection, defaecation frequency and body mass index. All these data can be derived from a questionnaire, including defaecation frequency: the Spearman correlation between reported and recorded defaecation frequency in our previous study was 0.84 (*P*<0.01). However, the complexity of the physiology of BA also requires experimental studies to understand better the effect of dietary factors on the DC/CDC ratio.
